# Supplementary material for: The immune landscape of human thymic epithelial tumors
Source: Nat Commun. 2022 Sep 17;13:5463. doi: 10.1038/s41467-022-33170-7 (PMC9482639; doi:10.1038/s41467-022-33170-7)
Supplement: Supplementary file 1 — Supplementary Information [file 41467_2022_33170_MOESM1_ESM.pdf]

The immune landscape of human thymic epithelial tumors

Zhongwei Xin[1,2,#], Mingjie Lin[1,2,#], Zhixing Hao[1,2,#], Di Chen[2,3], Yongyuan Chen[1,2], Xiaoke Chen[1,2], Xia Xu[4], Jinfan Li[4], Dang Wu[2,3], Ying Chai[1,*] and Pin Wu[1,2,*]

[1]Department of Thoracic Surgery, The Second Affiliated Hospital, Zhejiang University School of Medicine, Zhejiang University, Hangzhou, 310009, China

[2]Key Laboratory of Tumor Microenvironment and Immune Therapy of Zhejiang Province, The Second Affiliated Hospital, Zhejiang University School of Medicine, Zhejiang University, Hangzhou, 310009, China

[3]Department of Oncology Radiotherapy, The Second Affiliated Hospital, Zhejiang University School of Medicine, Zhejiang University, Hangzhou, 310009, China

[4]Department of Pathology, The Second Affiliated Hospital, Zhejiang University School of Medicine, Zhejiang University, Hangzhou, 310009, China

[#]These authors contributed equally: Zhongwei Xin, Mingjie Lin, Zhixing Hao

*Corresponding authors: Ying Chai, chaiy@zju.edu.cn; Pin Wu, pinwu@zju.edu.cn

Supplementary Information

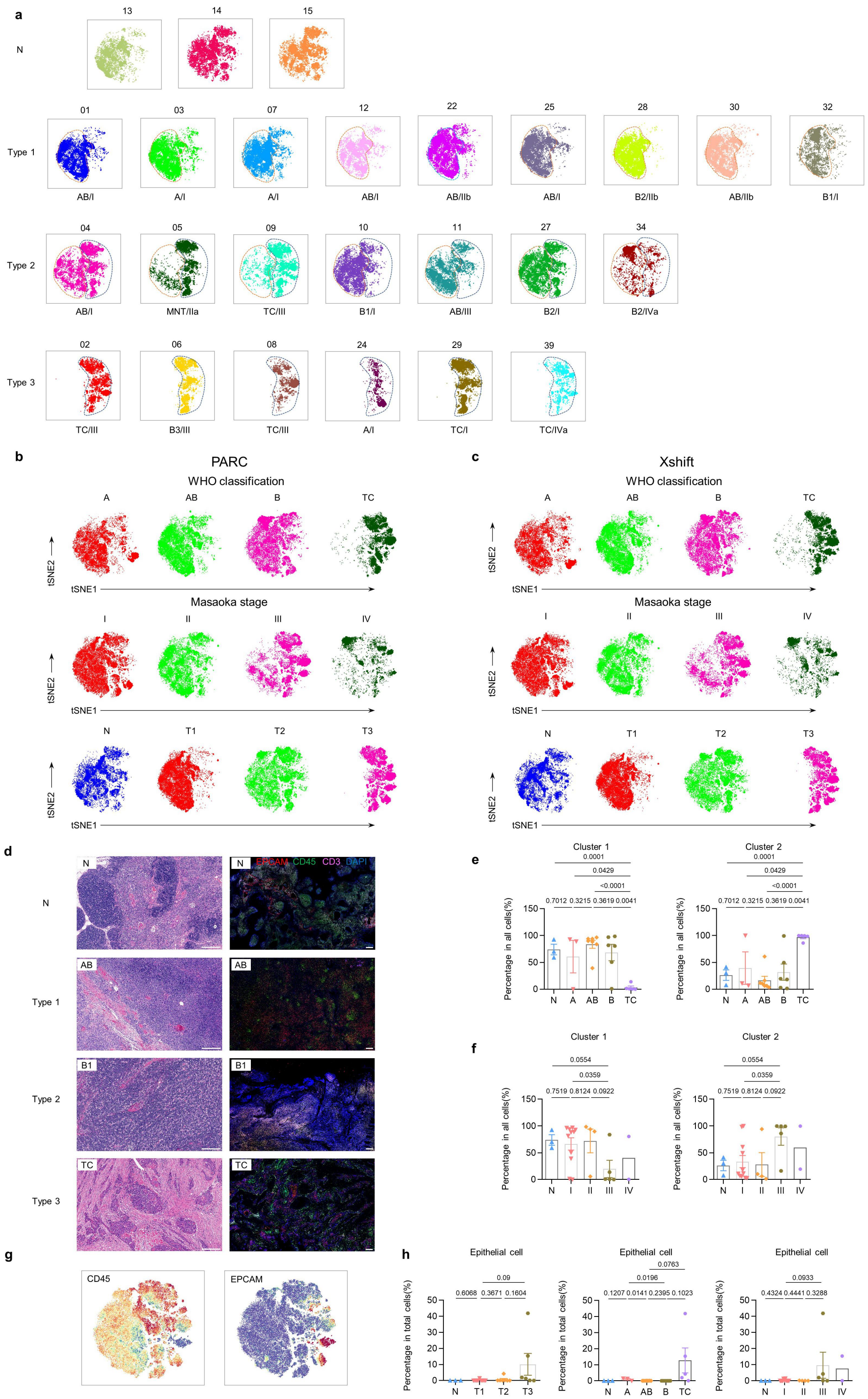

Supplementary Fig. 1 Reclassification of the human thymus and TETs.

(**a**) t-SNE plots of total cells from human TETs (n = 22) and the normal thymus (n=3) by CyTOF using a Phenograph clustering scheme, each color represents an independent sample (N, normal human thymus).

(**b-c**) t-SNE plots of total cells from human TETs (n = 22) and the normal thymus (n=3) by CyTOF using a PARC clustering scheme (**b**) or XSHIFT clustering scheme (**c**). Samples were grouped according to WHO histological subtype, Masaoka stage or reclassification and each color represents an independent group or stage. (N, normal human thymus; T1, type 1; T2, type 2; T3, type 3; the abbreviations below are consistent with these definitions).

(**d**) Representative HE and immunofluorescence (red for EpCAM, green for CD45, pink for CD3 and blue for DAPI) stained sections of TETs and the normal human thymus (scale bar: 200 μm). (TC, thymic carcinoma). Experiment was performed in three independent samples for each group with similar results.

(**e-f**) Bar plots showing the frequencies of cluster 1 and cluster 2 subsets among the WHO histological subtypes of samples (n=3, 3, 7, 6 and 5 for N, A, AB, B and TC, respectively) (**e**) or Masaoka stages of samples (n=3, 11, 4, 5 and 2 for N, I, II, III and IV, respectively) (**f**). (Data are presented as the mean ± s.e.m. *P* values in the figure were determined by an unpaired two-tailed Student's t test).

(**g**) t-SNE analysis of total cells from all samples colored by the relative expression of CyTOF markers (CD45 and EPCAM).

(**h**) Bar plots showing the frequencies of epithelial cell subset among the reclassifications of samples (n=3, 9, 7 and 6 for N, T1, T2 and T3, respectively)， WHO histological subtypes of samples (n=3, 3, 7, 6 and 5 for N, A, AB, B and TC, respectively) and Masaoka stages of samples (n=3, 11, 4, 5 and 2 for N, I, II, III and IV, respectively). (Data are presented as the mean ± s.e.m. *P* values in the figure were determined by an unpaired two-tailed Student's t test).

Source data are provided as a Source Data file.

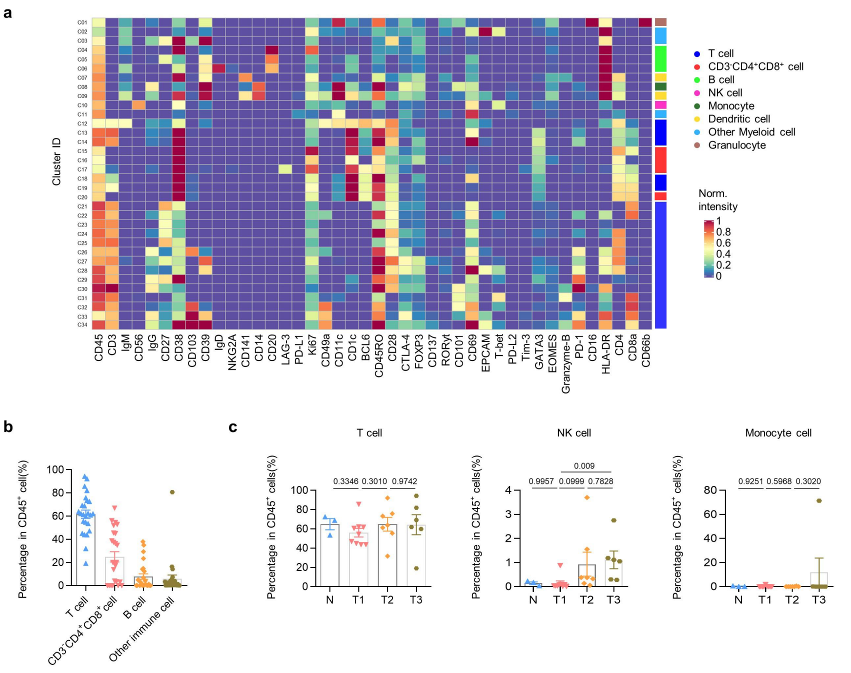

Supplementary Fig. 2. CD45^{+} immune cell subclustering by CyTOF.

(**a**) Heatmap showing relative normalized protein expression for every cluster of CD45^{+} cells from CyTOF, normalized total matrix.

(**b**) Bar plot showing the frequencies of different cell types (n=25. Data are presented as the mean ± s.e.m.).

(**c**) Bar plots showing the frequencies of the immune cell subsets among the four groups of samples (n=3, 9, 7 and 6 for N, T1, T2 and T3, respectively. Data are presented as the mean ± s.e.m. *P* values in the figure were determined by an unpaired two-tailed Student's t test).

Source data are provided as a Source Data file.

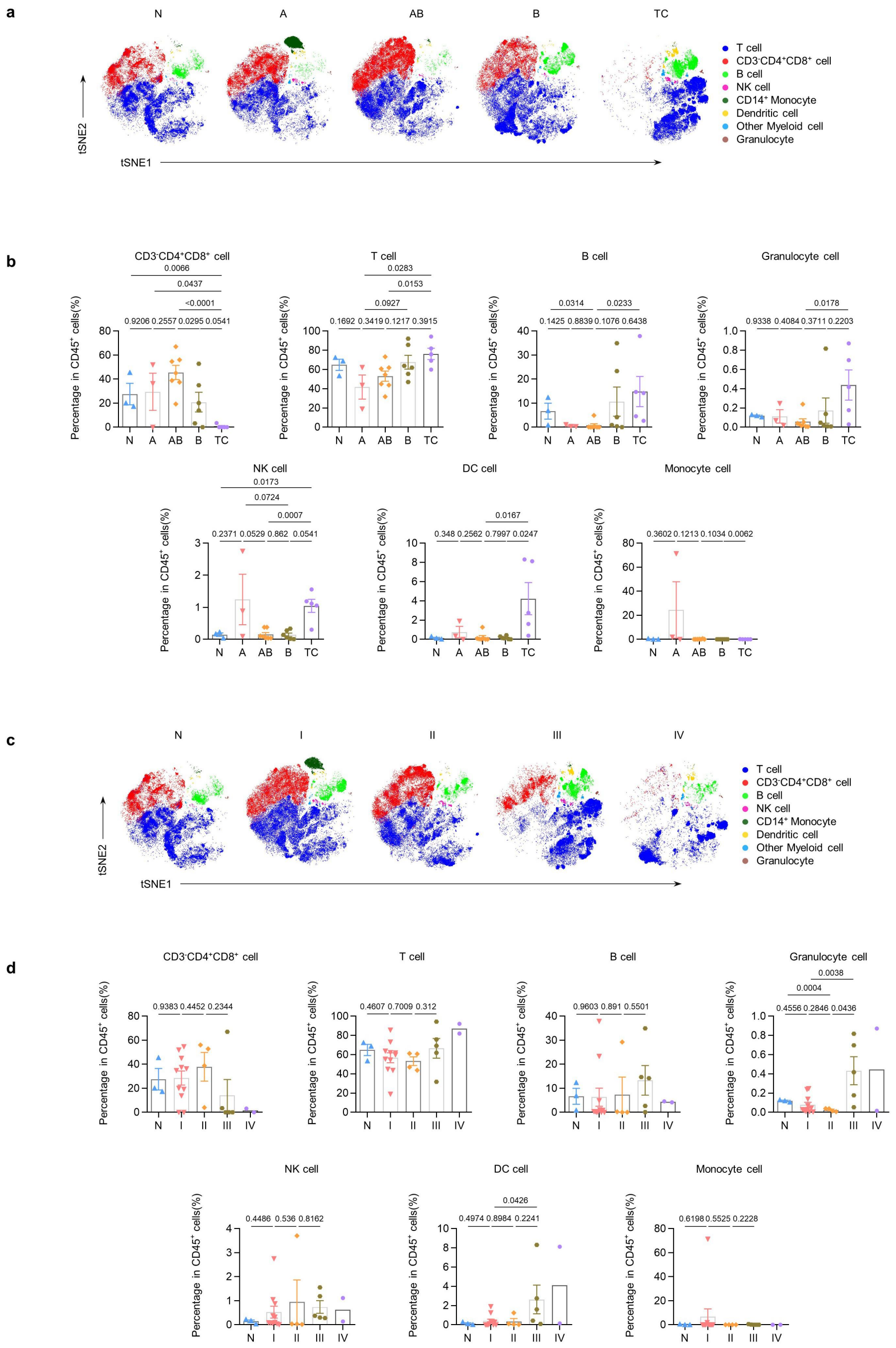

Supplementary Fig. 3. Differences in the proportion of immune cell subsets among WHO histologic subtypes and Masaoka stages from CyTOF.

(**a**) t-SNE plots of immune cells from samples of each WHO histological subtype (N, A, AB, B, TC) by CyTOF using a Phenograph clustering scheme, colored by cell type.

(**b**) Bar plots showing the frequencies of the main immune cell subsets among the WHO histological subtypes of samples (n=3, 3, 7, 6 and 5 for N, A, AB, B and TC, respectively. Data are presented as the mean ± s.e.m. *P* values in the figure were

determined by an unpaired two-tailed Student's t test).

(**c**) t-SNE plots of immune cells from samples of each Masaoka stage (N, I, II, III, IV) by CyTOF using a Phenograph clustering scheme, colored by cell type.

(**d**) Bar plots showing the frequencies of the main immune cell subsets among the Masaoka stages of samples (n=3, 11, 4, 5 and 2 for N, I, II, III and IV, respectively. Data are presented as the mean ± s.e.m. *P* values in the were determined by an unpaired two-tailed Student's t test).

Source data are provided as a Source Data file.

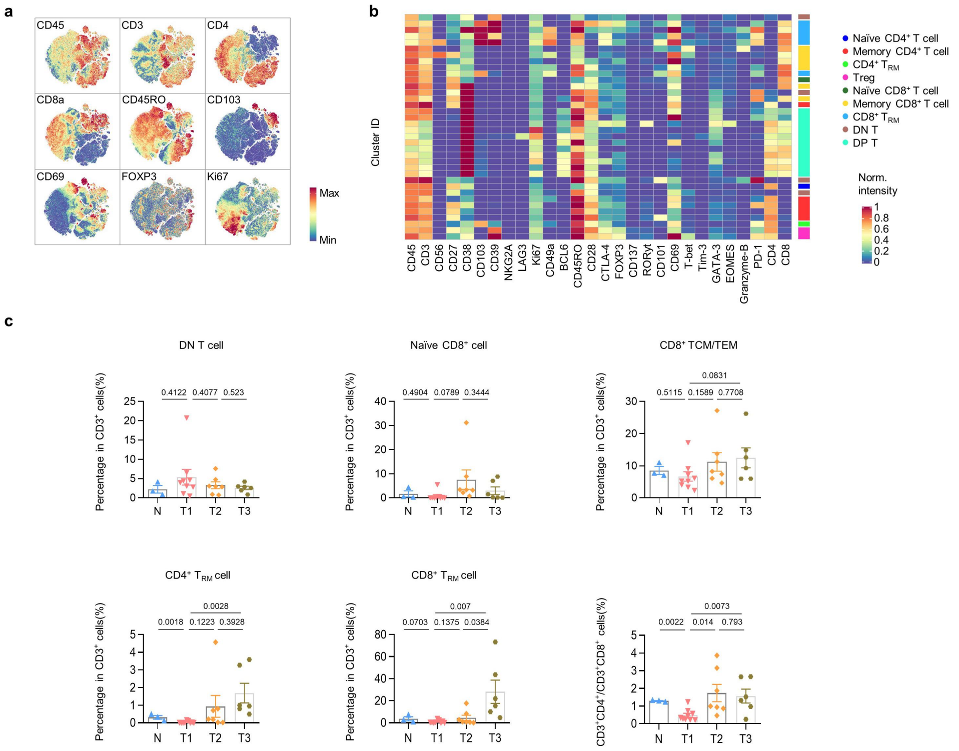

Supplementary Fig. 4. CD3$^+$ T cell subclustering by CyTOF.

(**a**) t-SNE analysis of CD3$^+$ T cells from all samples colored by the relative expression of CyTOF markers (CD45, CD3, CD4, CD8a, CD45RO, CD103, CD69, FOXP3 and Ki67).

(**b**) Heatmap showing relative normalized protein expression for every cluster of CD45$^+$ CD3$^+$ T cells from CyTOF, normalized total matrix.

(**c**) Bar plots showing the frequencies of T-cell subsets among the four groups of samples (n=3, 9, 7 and 6 for N, T1, T2 and T3, respectively. Data are presented as the mean ± s.e.m. *P* values in the figure were determined by an unpaired two-tailed Student's t test).

Source data are provided as a Source Data file.

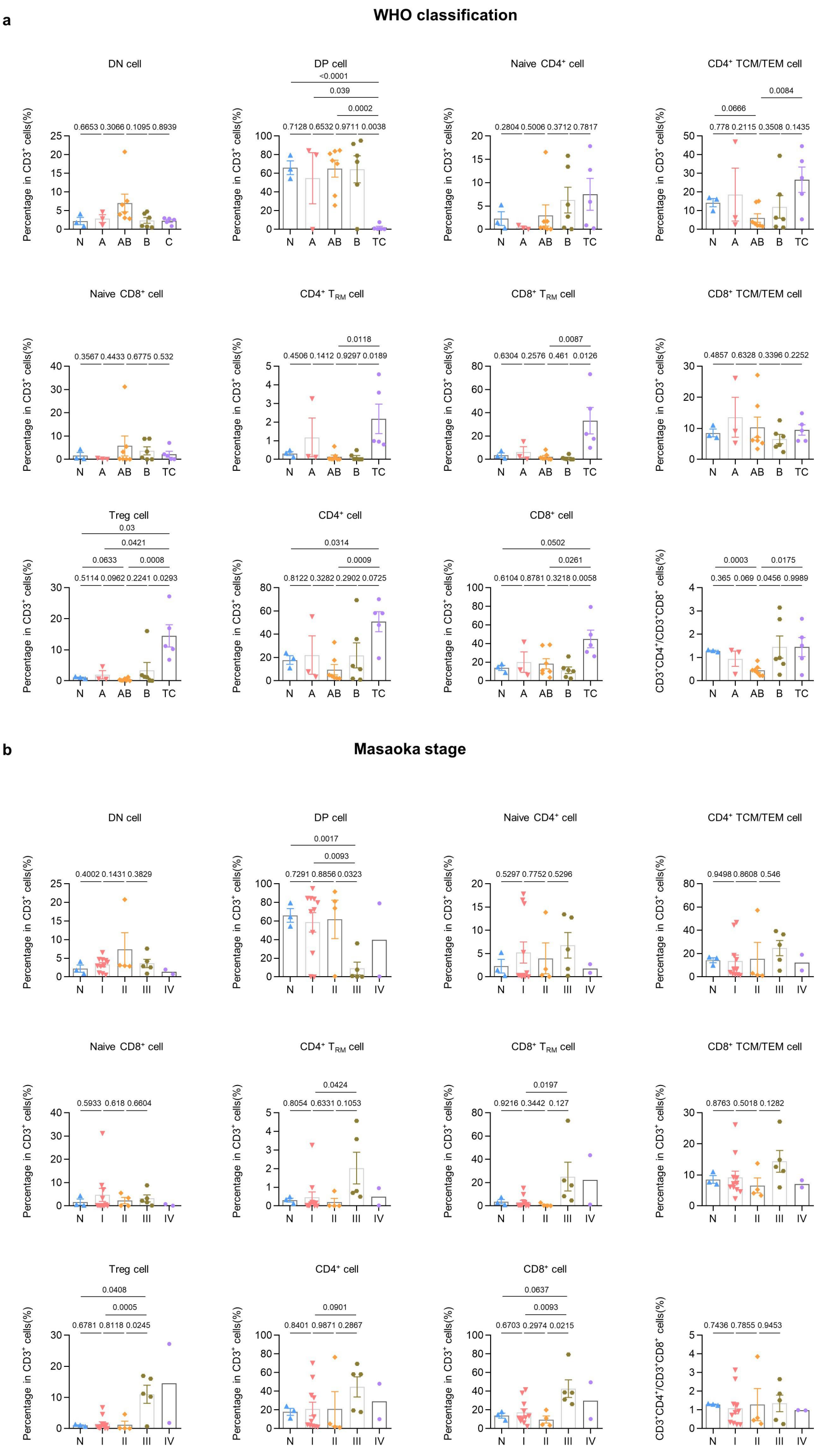

Supplementary Fig. 5. Differences in the proportion of T-cell subsets among WHO histologic subtypes and Masaoka stages from CyTOF.

(**a**) Bar plots showing the frequencies of the main T-cell subsets among the WHO histological subtypes of samples (n=3, 3, 7, 6 and 5 for N, A, AB, B and TC,

respectively. Data are presented as the mean ± s.e.m. *P* values in the figure were determined by an unpaired two-tailed Student's t test).

(**b**) Bar plots showing the frequencies of the main T-cell subsets among the Masaoka stages of samples (n=3, 11, 4, 5 and 2 for N, I, II, III and IV, respectively. Data are presented as the mean ± s.e.m. *P* values in the figure were determined by an unpaired two-tailed Student's t test).

Source data are provided as a Source Data file.

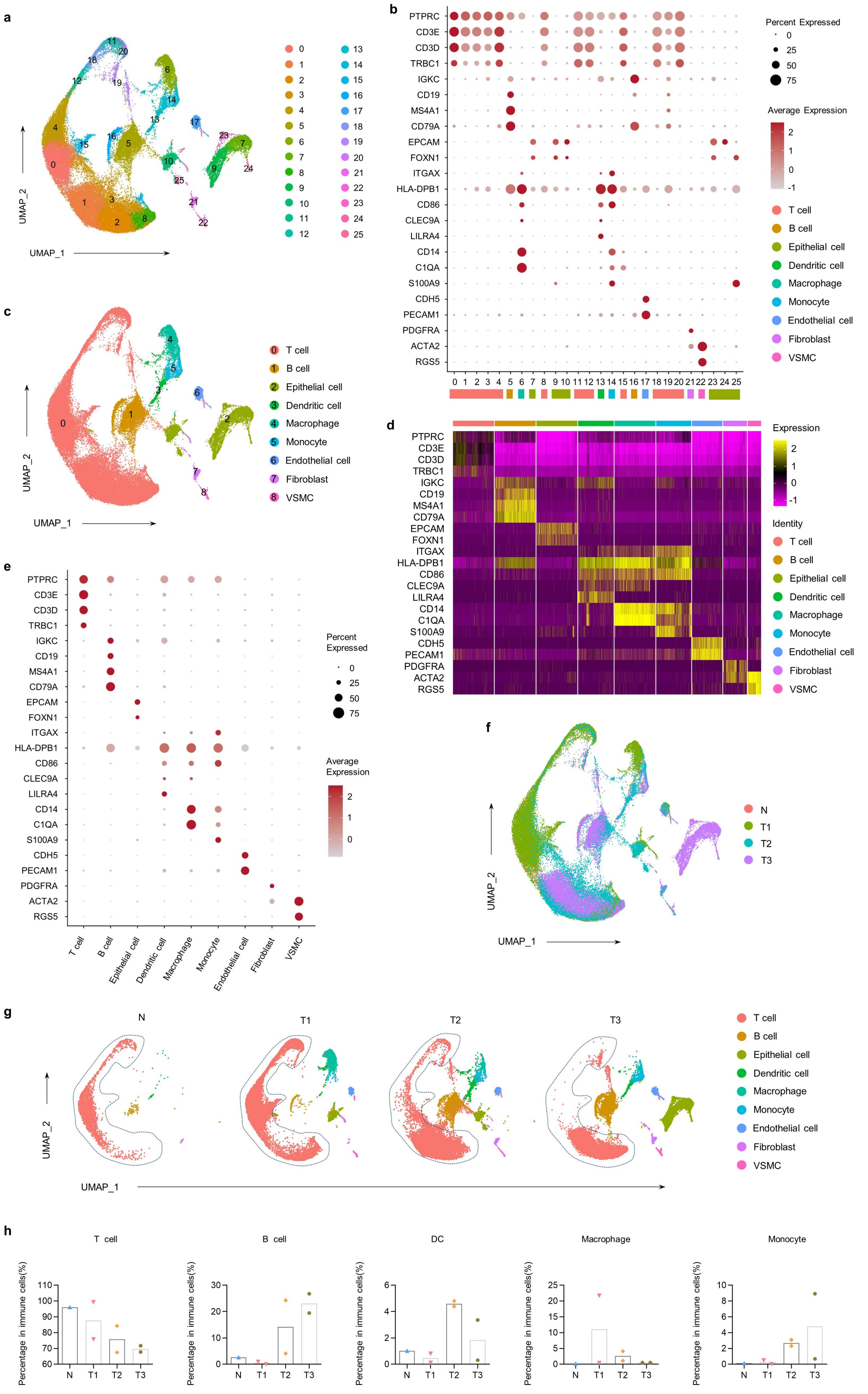

Supplementary Fig. 6. scRNA-seq revealed the cell composition of normal thymus and TET samples.

(**a**) UMAP of total cells from the normal human thymus (n=1) and TET samples (n=6), colored by the cell cluster.

(**b**) Dot plot of marker gene expression in each cluster of total cells. Here and in later figures, the color represents the maximum normalized mean expression of marker genes in each cell subgroup, and the size indicates the proportion of cells expressing marker genes.

(**c**) UMAP visualization of the major immune cellular composition from the normal human thymus (n=1) and TET samples (n=6), colored by the identified cell type. (VSMC, vascular smooth muscle cells).

(**d**) Heatmap and (**e**) Dot plot presenting the marker gene expression level among the identified cell types.

(**f**) Same UMAP plot as (**c**), colored by groups.

(**g**) Same UMAP plot of the cellular composition from samples of each group, colored by the identified cell type same as (**c**).

(**h**) Bar plots showing the frequencies of the main immune cell subsets among the four groups of samples from scRNA-seq (n=1, 2, 2 and 2 for N, T1, T2 and T3, respectively). Source data are provided as a Source Data file.

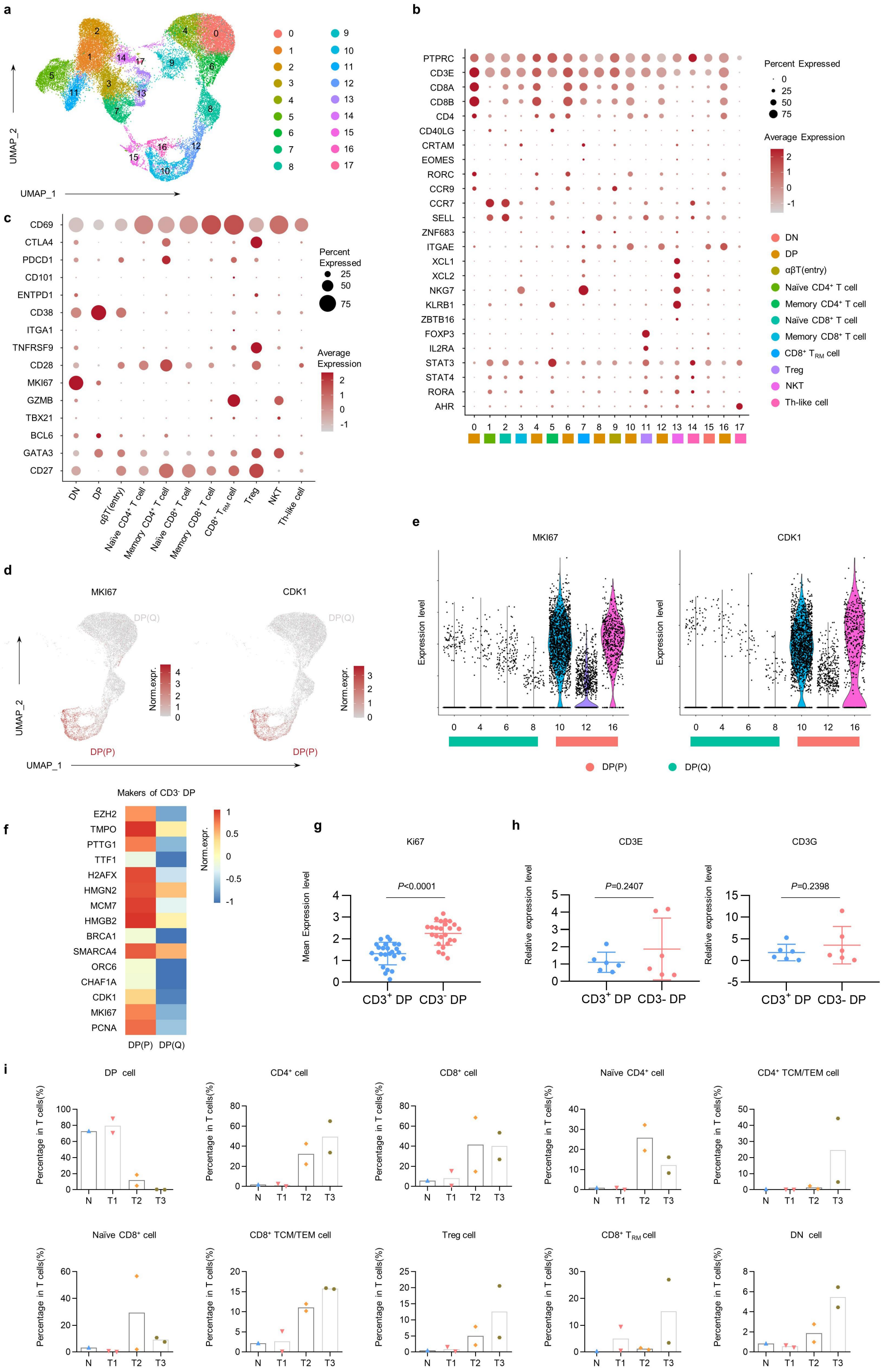

Supplementary Fig. 7. T cell subclustering by scRNA-seq.

(**a**) UMAP plot of T cells from the normal human thymus (n=1) and TET samples (n=6),

colored by the cell cluster.

(**b**) Dot plot of marker gene expression in each cluster of T cells.

(**c**) Dot plot for function related gene expression in each T-cell subset.

(**d**) UMAP plot of DP cells from the normal human thymus (n=1) and TET samples (n=6), colored by the gene expression (MKI67 and CDK1). (DP, double-positive T; P, proliferating; Q, quiescent).

(**e**) Violin plots showing the expression of the MKI67 and CDK1 genes among different cluster of DP cells.

(**f**) Heatmap showing the normalized expression of marker genes of $CD3^-$ DP cells within two subsets (defined by scRNA-seq) of DP cells, normalized per row.

(**g**) Bar plot showing the protein expression of Ki67 between $CD3^+$ DP cells and $CD3^+$ DP cells from CyTOF (n=25 for each group. Data are presented as the mean ± s.e.m. *P* values were determined by a paired two-tailed Student's t test).

(**h**) Bar plots showing the gene expression of CD3E and CD3G between $CD3^+$ DP cells and $CD3^+$ DP cells sorted by flow cytometry (n=6 for each group. Data are presented as the mean ± s.e.m. *P* values were determined by a paired two-tailed Student's t-test).

(**i**) Bar plots showing the frequencies of the T-cell subsets among the four groups of samples from scRNA-seq (n=1, 2, 2 and 2 for N, T1, T2 and T3, respectively).

Source data are provided as a Source Data file.

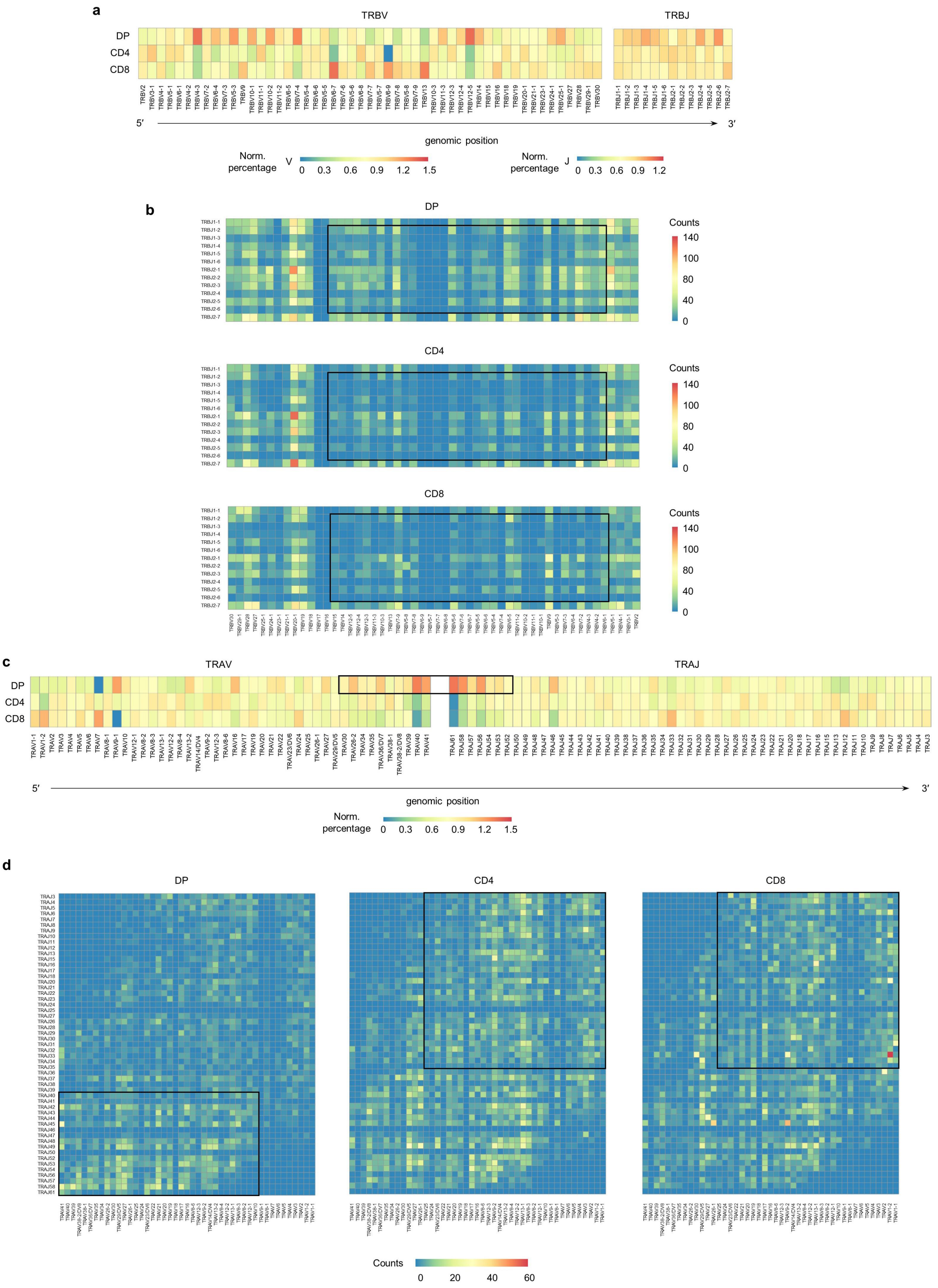

Supplementary Fig. 8. Bias in VJ gene usage and pairing during T cell development.

(**a**) Heatmap showing the proportion of each TCRβ V and J gene segment present in DP, $CD4^+$ T cells and $CD8^+$ T cells, normalized per column.

(**b**) Heatmap showing the frequency of V-J gene pairs at the TCRβ locus of DP, $CD4^+$ T cells and $CD8^+$ T cells.

(**c**) Same scheme as in (**a**) applied to TCRα V and J gene segments, normalized per column.

(**d**) Same scheme as in (**b**) applied to TCRα V-J gene pairs.

Source data are provided as a Source Data file.

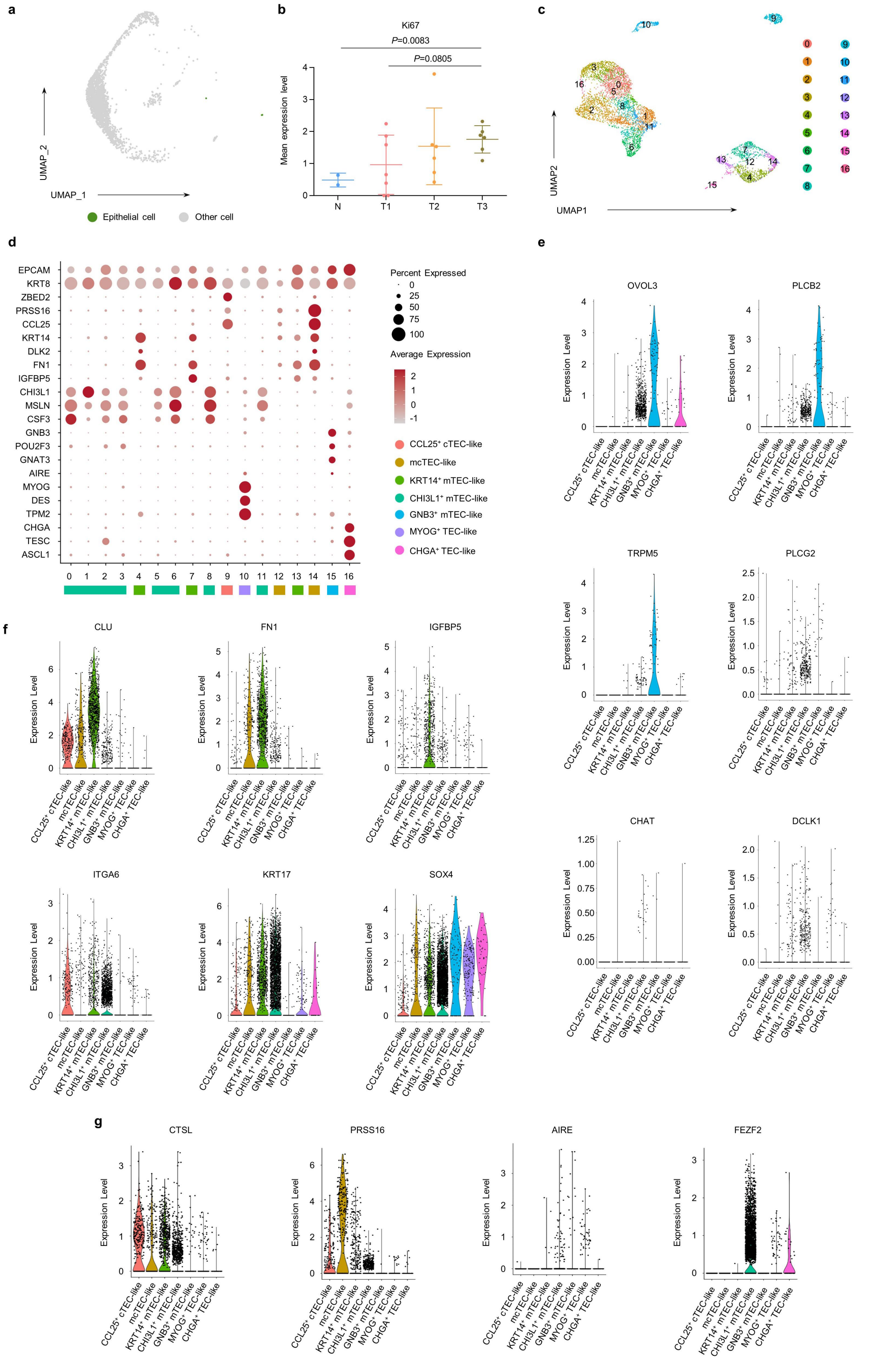

Supplementary Fig. 9. Discrepancy of marker genes expression among different epithelial cell subsets.

(**a**) Same UMAP plot as (**Figure S6c**) of the cellular composition from samples of the normal thymus, colored by the cell types of epithelials cell and other cells.

(**b**) Bar plot showing the protein expression of Ki67 on epithelial cells among groups by CyTOF (n=2, 7, 6 and 6 for N, T1, T2 and T3, respectively. Data are presented as the mean ± s.e.m. *P* values were determined by an unpaired two-tailed Student's t test).

(**c**) UMAP plot of epithelial cells from the normal human thymus (n=1) and TET samples (n=6), colored by the cell cluster.

(**d**) Dot plot of the marker gene expression in each cluster of epithelial cells.

(**e-g**) Violin plots showing the expression level of marker genes for tuft-like mTEC in the normal thymus (**e**), KRT14 mTEC(I) in the normal thymus (**f**) and genes associated with positive and negative selection functions (**g**) among different epithelial cell subpopulations. (mTEC, medullar thymic epithelial cells).

Source data are provided as a Source Data file.

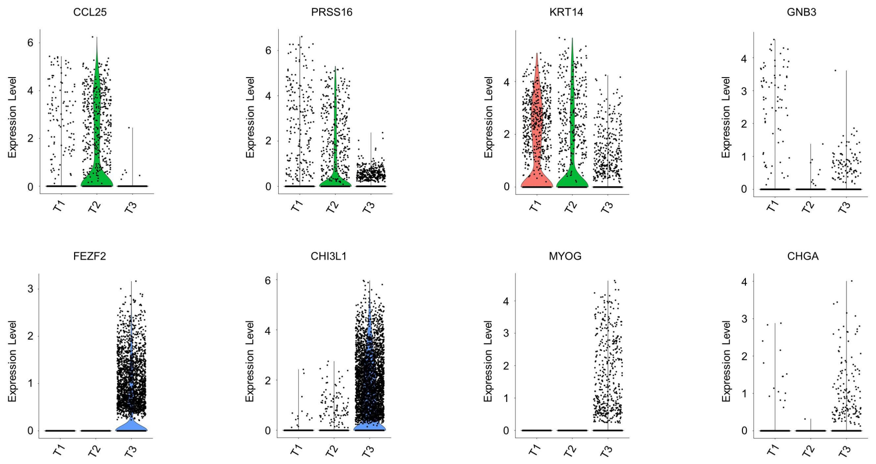

Supplementary Fig. 10. Discrepancy of marker genes expression in epithelial cells among the three TET tumor types.

Violin plots showing the expression of the TEC marker genes in epithelial cells among different types of TETs.

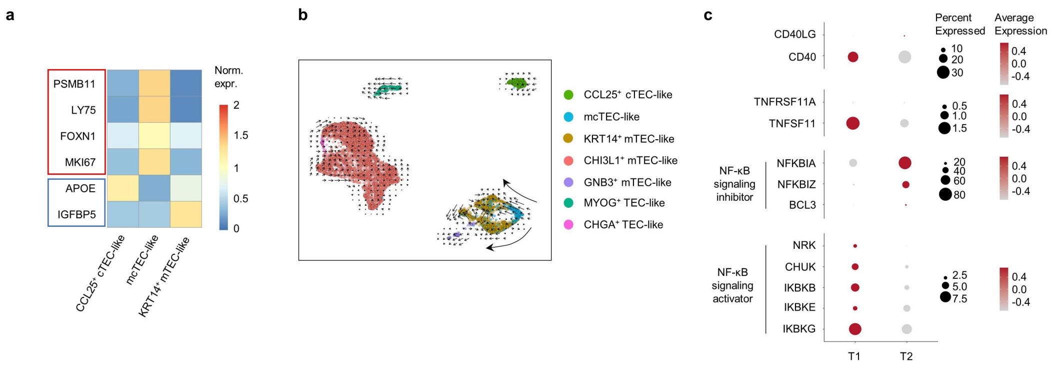

Supplementary Fig. 11. Potential developmental relationship among epithelial cell subsets.

(**a**) Heatmap showing the expression of marker genes related to thymic epithelial progenitor cells (TEPCs) within cTEC-like cells, mcTEC-like cells and KRT14+ mTEC-like cells, normalized per row. Genes with high expression in TEPC indicated by the red frame and low expression in TEPC indicated by the blue frame.

(**b**) RNA velocity analysis of epithelial cell subsets visualized on the UMAP same as (**Figure 5e**), colored by the identified cell subpopulation.

(**c**) Dot plot depicting the relative expression levels of selected NF-κB signaling pathway genes in mcTEC-like cells from type 1 and type 2 TETs.

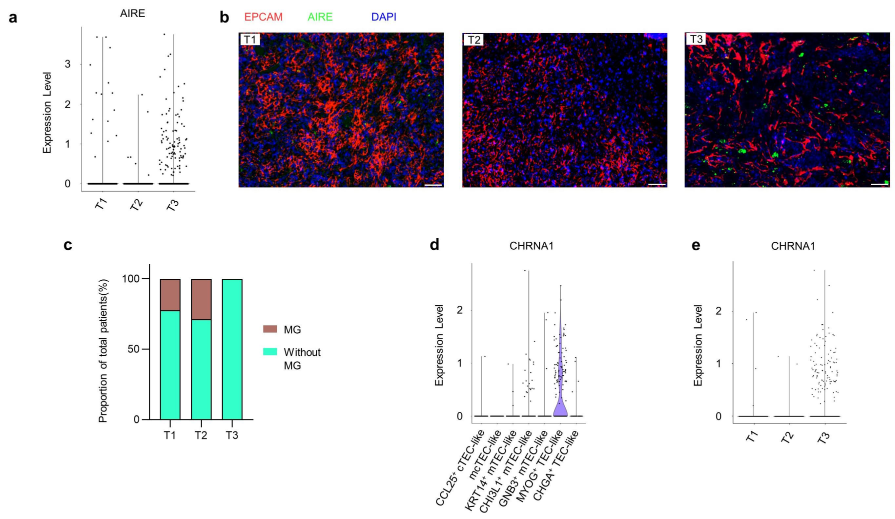

Supplementary Fig. 12. Association between the reclassification and MG incidence.

(**a**) Violin plot showing the expression level of *AIRE* gene in epithelial cells among different types of TETs.

(**b**) Representative immunofluorescence (IF) staining images showing EPCAM (red), AIRE (green) and DAPI (nuclei, blue) in TET samples. (scale bar: 50 μm). Experiment was performed in three independent samples for each group with similar results.

(**c**) Bar plot showing the proportion of patients with or without MG in each group (n=9, 7 and 6 for T1, T2 and T3 respectively), color represents the patient with or without MG. (MG, myasthenia gravis).

(**d**) Violin plot showing the expression level of *CHRNA1* gene among different epithelial cell subpopulations.

(**e**) Violin plot showing the expression level of *CHRNA1* gene in epithelial cells among different types of TETs.

Source data are provided as a Source Data file.

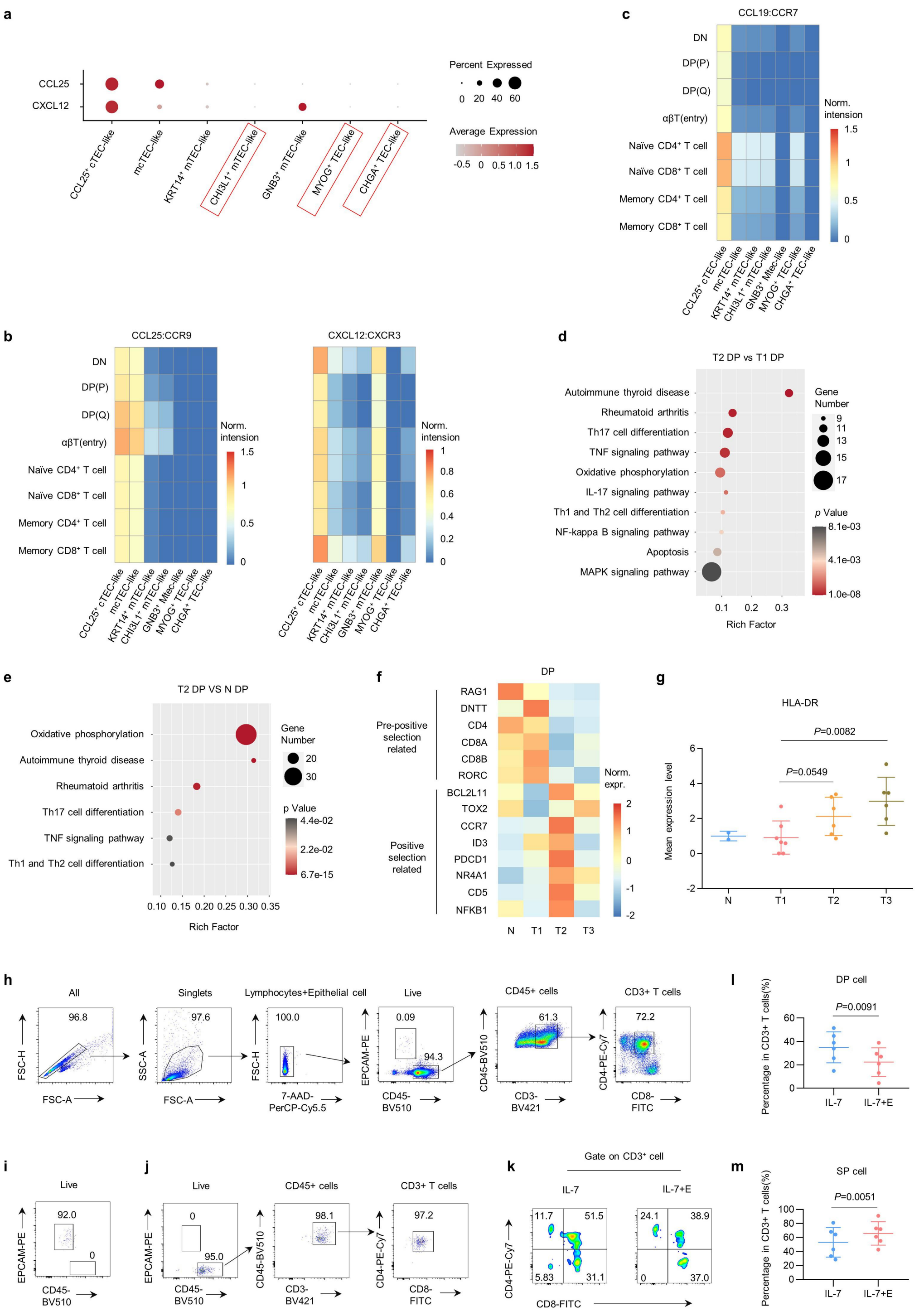

Supplementary Fig. 13. Effect of epithelial cells on T cell development of TETs.

(**a**) Dot plot of the expression of *CCL25* and *CXCL12* genes in epithelial cell subpopulations and epithelial subsets in type 3 TETs indicated by the red frame.

(**b-c**) Heat maps of chemokine interactions among subpopulations of T cells and epithelial cells, chemokine expressed by the epithelial cell type and the cognate receptor by the T-cell subset. It is normalized total matrix. (DN, double-negative T cells;

DP, double-positive T cells; P, proliferating; Q, quiescent).

(**d-e**) KEGG pathway analysis of genes that were significantly ($P < 0.01$) upregulated in DP cells of type 2 TETs compared to the type 1 TETs (**d**) and the normal thymus (**e**). (DP, double-positive T cells; T2, type 2; N, normal thymus).

(**f**) Heatmap showing the normalized expression of preselection- and selection-associated genes within DP cells in each group by scRNA-seq, normalized per row.

(**g**) Bar plot showing the protein expression of HLA-DR on epithelial cells among groups by CyTOF (n=2, 7, 6 and 6 for N, T1, T2 and T3, respectively. Data are presented as the mean ± s.e.m. *P* values were determined by an unpaired two-tailed Student's t test).

(**h**) Representative gating strategy of flow cytometry used for sorting epithelial cell and $CD3^+$ DP cells from TET samples.

(**i-j**) Representative flow cytometric plots of quality control for sorted epithelial cells (**i**) and $CD3^+$ DP cells (**j**) from TET samples.

(**k**) Representative flow cytometric plot of $CD3^+CD4^-CD8^-$ T cells, $CD3^+CD4^+$ T cells, $CD3^+CD8^+$ T cells and $CD3^+CD4^+CD8^+$ T cells after epithelial cells and $CD3^+$ DP cells co-culture in vitro for 8 days. (E, epithelial cell).

(**l-m**) Bar plots showing the frequencies of DP cells (**l**) and SP cells (**m**) between the control group (IL-7) and co-culture group (IL-7 with epithelial cell) (n= 6 for each group. Data are presented as the mean ± s.e.m. *P* values were determined by a paired two-tailed Student's t test). (DP cell, $CD3^+CD4^+CD8^+$ T cell; SP, $CD3^+CD4^+$ and $CD3^+CD8^+$ T cell).

Source data are provided as a Source Data file.

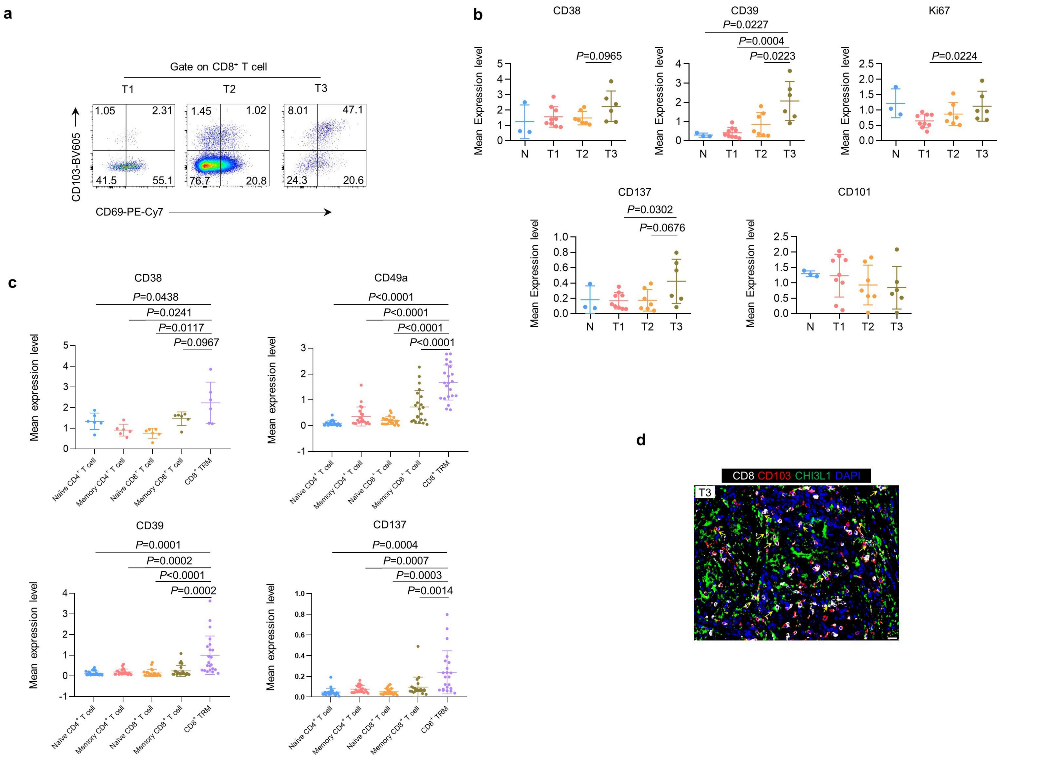

Supplementary Fig. 14. Enrichment and activation of CD8$^+$ T$_{RM}$ cells in type 3 TETs.

(**a**) Representative flow cytometric plot of CD8$^+$ T$_{RM}$ (CD69$^+$ CD103$^+$ CD8$^+$ T) cells from TETs of each type.

(**b**) Bar plots showing the protein expression level of CD38, CD39, Ki67, CD137 and CD101 on CD8$^+$ T$_{RM}$ cells among groups from CyTOF (n=3, 9, 7 and 6 for N, T1, T2 and T3, respectively. Data are presented as the mean ± s.e.m. *P* values were determined by an unpaired two-tailed Student's t test).

(**c**) Bar plots showing the protein expression level of CD38 on T-cell subsets of type3 TETs (n=6 for each group), CD49a, CD39 and CD137 on T-cell subsets of TET samples from CyTOF (n=22 for each group. Data are presented as the mean ± s.e.m. *P* values were determined by a paired two-tailed Student's t test).

(**d**) Representative IF staining images showing CD8 (white), CD103 (red), CHI3L1 (green) and DAPI (nuclei, blue) in type 3 TET samples. The interactions between CHI3L1$^+$ epithelial cells and T$_{RM}$ cells are indicated by yellow arrows. Scale bar: 20 μm. Experiment was performed in three independent samples with similar results.

Source data are provided as a Source Data file.

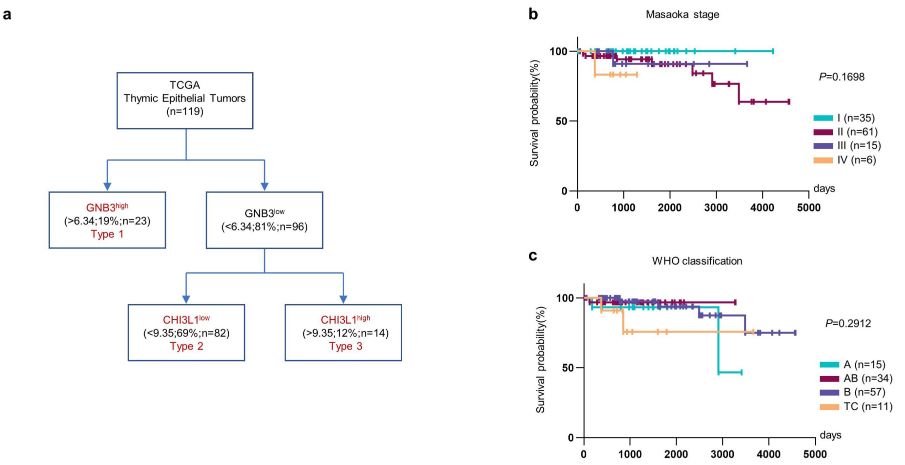

Supplementary Fig. 15. Prognosis of patients with TETs in the TCGA cohort.

(**a**) Illustration of the TET subtype classification tree.

(**b**) Overall survival (OS) examined in each of Masaoka stage of TETs in the TCGA cohort, *P* value is calculated using the log-rank test.

(**c**) Overall survival (OS) examined in each type of WHO classification of TETs in the TCGA cohort, *P* value is calculated using the log-rank test.

Source data are provided as a Source Data file.

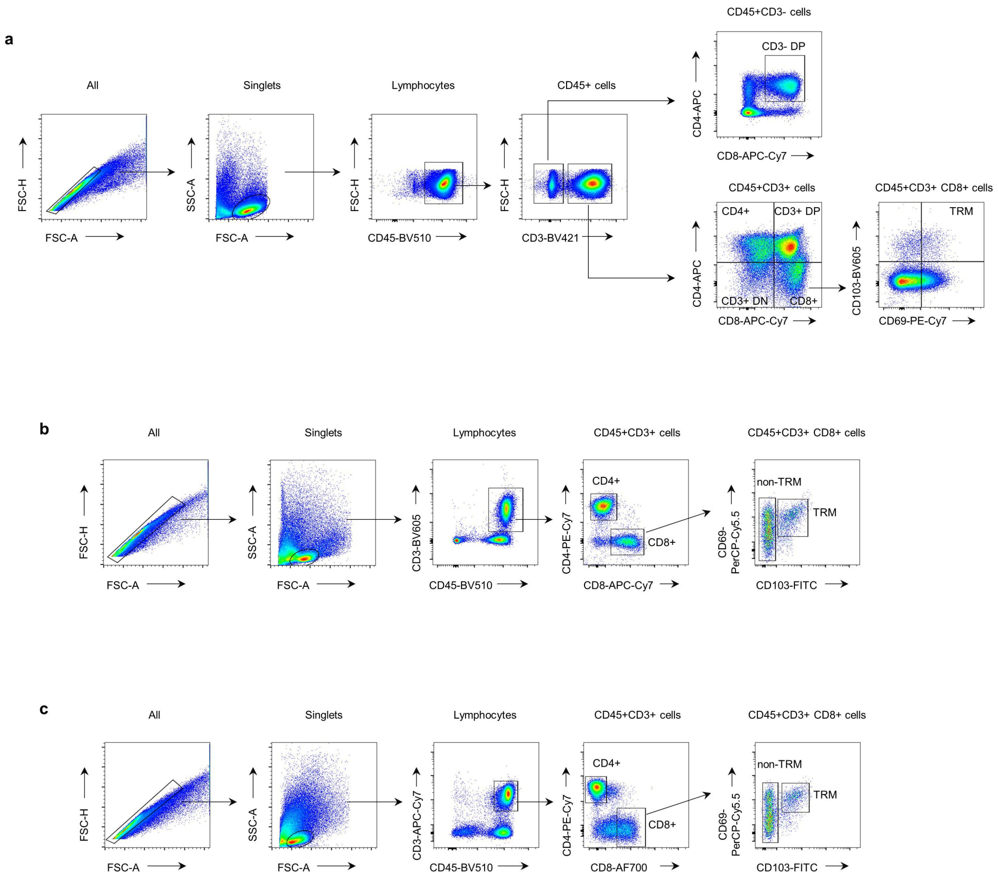

Supplementary Fig. 16. Gating strategies used for flow cytometry.

(**a**) Gating strategy for flow cytometric analysis presented on Fig. 1l, 2h and Supplementary Fig. 14a. $CD3^+$ T ($CD45^+CD3^+$) cell; $CD3^-$ DP ($CD45^+CD3^-CD4^+CD8^+$) cell; $CD4^+$ T ($CD45^+CD3^+CD4^+CD8^-$) cell; $CD8^+$ T ($CD45^+CD3^+CD8^+CD4^-$) cell; $CD3^+$ DP ($CD45^+CD3^+CD4^+CD8^+$) cell; $CD3^+$ DN ($CD45^+CD3^+CD4^-CD8^-$) cell; $CD8^+$ T_{RM} ($CD45^+CD3^+CD8^+CD69^+CD103^+$) cell.

(**b-c**) Gating strategy for flow cytometric analysis presented on Fig.6e (**b**) and Fig.6i, m (**c**). $CD4^+$ T ($CD45^+CD3^+CD4^+CD8^-$) cell; $CD8^+$ non-T_{RM} ($CD45^+CD3^+CD8^+CD69^{+/-}$ $CD103^-$) cell; $CD8^+$ T_{RM} ($CD45^+CD3^+CD8^+CD69^+CD103^+$) cell.

Supplementary Table 1: Antibodies for CyTOF.

list	label	marker	clone	Vendor	CAT	dilution	Staining
1	89Y	CD45	HI30	BioLegend	304002	100	Surface
2	115In	CD3	UCHT1	BioXcell	BE0231	200	Surface
3	139La	IgM	MHM-88	BioLegend	314502	200	Surface
4	141Pr	CD56	NCAM16.2	BD	559043	800	Surface
5	142Nd	IgG	G18-145	BD	555784	25	Surface
6	143Nd	CD27	O323	BioLegend	302802	100	Surface
7	144Nd	CD38	HIT2	BioLegend	303502	100	Surface
8	145Nd	CD103	B-Ly7	eBioscience	14-1038-82	200	Surface
9	146Nd	CD39	A1	BioLegend	328202	100	Surface
10	147Sm	IgD	IA6-2	BioLegend	348202	400	Surface
11	148Nd	NKG2A	131411	RD	MAB1059	50	Surface
12	149Sm	CD141	M80	BioLegend	344102	200	Surface
13	150Nd	CD14	M5E2	BioLegend	301810	50	Surface
14	151Eu	CD20	2H7	RD	MAB9575-100	200	Surface
15	152Sm	LAG3	874501	RD	MAB23193	100	Surface
16	153Eu	PD-L1	29E.2A3	BioLegend	329702	400	Surface
17	154Sm	Ki67	SolA15	Thermo	14-5698-82	100	Intracellular
18	155Gd	CD49a	TS2/7	BioLegend	328302	200	Surface
19	156Gd	CD11c	BU15	BioLegend	337202	400	Surface
20	157Gd	CD1c	L161	BioLegend	331502	100	Surface
21	158Gd	BCL6	K112-91	BD	561520	100	Surface
22	159Tb	CD45RO	UCHL1	BioLegend	304202	200	Surface
23	160Gd	CD28	CD28.2	BioXcell	BE0291	100	Surface
24	161dy	CTLA-4	L3D10	BioLegend	349902	100	Surface
25	162Dy	FoxP3	PCH101	eBioscience	14-4776-82	50	Intracellular
26	163Dy	CD137	4B4-1	BioLegend	309802	100	Surface
27	164Dy	RORγt	600214	RD	MAB6109	50	Intracellular
28	165Ho	CD101	BB27	BioLegend	331010	100	Surface
29	166Er	CD69	FN50	BioLegend	310902	200	Surface
30	167Er	EpCAM	9C4	BioLegend	324202	200	Surface
31	168Er	T-bet	4B10	BioLegend	644802	100	Intracellular
32	169Tm	PD-L2	24F.10C12	BioLegend	329610	400	Surface
33	170Er	Tim-3	F38-2E2	BioLegend	345004	50	Surface
34	171Yb	GATA-3	TWAJ	Invitrogen	50-9966-42	50	Intracellular
35	172Yb	EOMES	644730	RD	MAB6166	25	Intracellular
36	173Y	granzyme B	GB11	Fluidigm	3173006B	200	Intracellular
37	174Yb	PD-1	EH12.2H7	BioLegend	329926	50	Surface
38	175Lu	CD16	3G8	BioLegend	302014	100	Surface
39	176Yb	HLA-DR	L243	BioLegend	307612	200	Surface
40	197Au	CD4	RPA-T4	BioLegend	300516	400	Surface
41	198Pt	CD8	RPA-T8	BioLegend	301018	400	Surface

42	209Bi	CD66b	M1/70	BioLegend	101202	800	Surface

Supplementary Table 2: Patient characteristics with correlative assays performed.

Patient#	Tissue Type	Gender	Age	WHO classification	Masaoka stage	MG	CyTOF	scRNA-seq	IF	FCM	Co-culture
01	TETs	Male	56	AB	I	n	y	y	n	y	n
02	TETs	Male	69	TC	III	n	y	y	n	y	n
03	TETs	Female	72	A	I	n	y	y	n	y	n
04	TETs	Male	55	AB	I	y	y	y	n	y	n
05	TETs	Female	67	MNT	IIa	n	y	y	n	y	n
06	TETs	Male	44	B3	III	n	y	y	n	n	n
07	TETs	Female	31	A	I	n	y	n	y	n	n
08	TETs	Male	68	TC	III	n	y	n	y	n	n
09	TETs	Female	58	TC	III	n	y	n	y	n	n
10	TETs	Female	37	B1	I	n	y	n	n	n	n
11	TETs	Male	66	AB	III	n	y	n	y	n	n
12	TETs	Male	68	AB	I	n	y	n	y	n	n
13	Normal thymus	Female	27	\	\	n	y	n	y	n	n
14	Normal thymus	Female	46	\	\	n	y	n	y	n	n
15	Normal thymus	Female	34	\	\	n	y	n	n	n	n
16	TETs	Female	35	B2	III	n	n	n	y	n	n
17	TETs	Male	60	B2	I	n	n	n	n	n	n
18	TETs	Male	61	B2	III	y	n	n	n	n	n
19	TETs	Female	63	AB	I	n	n	n	y	n	n
20	TETs	Male	50	B2	III	n	n	n	n	y	n
21	TETs	Female	25	B3	III	n	n	n	n	y	n
22	TETs	Male	73	AB	IIb	y	y	n	y	y	n
23	TETs	Male	48	A	I	n	n	n	y	y	n
24	TETs	Male	68	A	I	n	y	n	y	y	n
25	TETs	Male	58	AB	I	n	y	n	y	y	n
26	TETs	Female	61	AB	I	n	n	n	y	y	n
27	TETs	Female	63	B2	I	y	y	n	y	y	n
28	TETs	Female	57	B2	IIb	n	y	n	y	y	n
29	TETs	Female	61	TC	I	n	y	n	y	y	n
30	TETs	Male	49	AB	IIb	n	y	n	y	y	n
31	TETs	Male	41	AB	I	n	n	n	y	y	n
32	TETs	Female	55	B1	I	y	y	n	y	y	n
33	TETs	Female	63	B2	I	y	n	n	y	y	n
34	TETs	Female	42	B2	IVa	n	y	n	y	y	y
35	TETs	Female	36	AB/B2	IIb	y	n	n	y	y	y
36	TETs	Female	35	B1	I	n	y	n	y	y	n

37	TETs	Female	55	TC	I	n	n	n	y	y	n
38	TETs	Female	51	B2	I	y	n	n	y	y	y
39	TETs	Female	51	TC	IVa	n	y	n	y	y	n
40	TETs	Female	46	B2	I	n	n	n	y	y	y
41	TETs	Female	49	B1	I	n	n	n	n	y	y
42	TETs	Male	45	TC	III	n	n	n	n	y	n
43	TETs	Male	58	TC	III	n	n	n	n	y	n
44	TETs	Female	63	B2	I	n	n	n	n	y	n
45	TETs	Female	50	B2	I	n	n	n	n	y	y

TETs: thymic epithelial tumours, TC: thymic carcinoma, MG: myasthenia gravis, CyTOF: mass cytometry, scRNA-seq: Single-cell RNA sequencing, IF: immunofluorescence, FCM: flow cytometry, Co-culture: in vitro co-culture

Supplementary Table 3: Sequencing statistics of each sample.

Sample	Estimated Number of Cells	Mean Reads per Cell	Median Genes per Cell	Reads Mapped to Genome	Reads Mapped Confidently to Genome	Reads Mapped Confidently to Intergenic Regions
N	2,845	168,118	1,293	95.1%	86.0%	1.8%
X-1	8,971	35,842	1,196	91.9%	85.2%	1.4%
X-2	10,824	27,398	1,710	93.3%	83.7%	0.8%
X-3	10,647	29,579	1,315	90.7%	79.5%	1.2%
X-4	5,219	69,720	1,660	93.4%	80.8%	0.9%
X-5	9,482	33,580	1,325	92.1%	82.6%	1.1%
X-6	7,645	36,548	1,337	92.9%	81.2%	0.9%

Sample	Reads Mapped Confidently to Intronic Regions	Reads Mapped Confidently to Exonic Regions	Reads Mapped Confidently to Transcriptome	Reads Mapped Antisense to Gene	Fraction Reads in Cells	Total Genes Detected
N	16.3%	67.9%	59.3%	4.1%	78.4%	25,015
X-1	8.1%	75.8%	66.8%	4.2%	88.3%	27,005
X-2	3.9%	79.1%	70.9%	3.6%	95.0%	31,514
X-3	5.8%	72.5%	64.4%	3.9%	89.8%	30,327
X-4	5.0%	75.0%	68.4%	2.4%	87.7%	28,431
X-5	7.9%	73.6%	66.7%	2.8%	67.0%	29,505
X-6	5.4%	74.9%	68.1%	2.7%	86.8%	26,441

Supplementary Table 4: Primer sequences.

ID	GENE	Forward Primer	Reverse Primer
1	CD3E	CCTCTTATCAGTTGGCGTTTGG	TTCAGTGACAGGTGATCCTCA
2	CD3G	TGGCCCAGTCAATCAAAGGAA	CAAGTCAGAAGTACCGAACCATC
3	GAPDH	ACATCATCCCTGCATCCACT	GTCCTCAGTGTAGCCCAAG

All the primers were purchased from Tsingke Biotechnology Co., Ltd.
