# Supplementary material for: The immune landscape of human thymic epithelial tumors
Source: Nat Commun. 2022 Sep 17;13:5463. doi: 10.1038/s41467-022-33170-7 (PMC9482639; doi:10.1038/s41467-022-33170-7)
Supplement: Supplementary file 3 — Description of Additional Supplementary Files [file 41467_2022_33170_MOESM3_ESM.pdf]

Description of Additional Supplementary Files

File Name: Supplementary Data 1
Description: **Cell subpopulation gene markers**, related to Supplementary Fig. 6. *P* values were determined by one-tailed likelihood-ratio test; p_val_adj were determined by one-tailed likelihood-ratio test followed by Bonferroni correction.

File Name: Supplementary Data 2
Description: **T cell subpopulation gene markers**, related to Figure 3 and Supplementary Fig. 7. *P* values were determined by one-tailed likelihood-ratio test; p_val_adj were determined by one-tailed likelihood-ratio test followed by Bonferroni correction.

File Name: Supplementary Data 3
Description: **Tumor cell subpopulation gene markers**, related to Figure 5 and Supplementary Fig. 9-10. *P* values were determined by one-tailed likelihood-ratio test; p_val_adj were determined by one-tailed likelihood-ratio test followed by Bonferroni correction.

File Name: Supplementary Data 4
Description: Ligand-receptor means for cell-cell interactions (from CellPhoneDB), related to Figure 6 and Supplementary Fig. 13.
