# Supplementary material for: The immune landscape of human thymic epithelial tumors
Source: Nat Commun. 2022 Sep 17;13:5463. doi: 10.1038/s41467-022-33170-7 (PMC9482639; doi:10.1038/s41467-022-33170-7)
Supplement: Supplementary file 8 — Reporting Summary [file 41467_2022_33170_MOESM8_ESM.pdf]

nature portfolio

Corresponding author(s): Pin Wu

Last updated by author(s): Aug 14, 2022

Reporting Summary

Nature Portfolio wishes to improve the reproducibility of the work that we publish. This form provides structure for consistency and transparency in reporting. For further information on Nature Portfolio policies, see our Editorial Policies and the Editorial Policy Checklist.

Statistics

For all statistical analyses, confirm that the following items are present in the figure legend, table legend, main text, or Methods section.

n/a	Confirmed	
☐	☒	The exact sample size (n) for each experimental group/condition, given as a discrete number and unit of measurement
☒	☐	A statement on whether measurements were taken from distinct samples or whether the same sample was measured repeatedly
☐	☒	The statistical test(s) used AND whether they are one- or two-sided<br>*Only common tests should be described solely by name; describe more complex techniques in the Methods section.*
☒	☐	A description of all covariates tested
☒	☐	A description of any assumptions or corrections, such as tests of normality and adjustment for multiple comparisons
☐	☒	A full description of the statistical parameters including central tendency (e.g. means) or other basic estimates (e.g. regression coefficient) AND variation (e.g. standard deviation) or associated estimates of uncertainty (e.g. confidence intervals)
☐	☒	For null hypothesis testing, the test statistic (e.g. F, t, r) with confidence intervals, effect sizes, degrees of freedom and P value noted<br>*Give P values as exact values whenever suitable.*
☒	☐	For Bayesian analysis, information on the choice of priors and Markov chain Monte Carlo settings
☒	☐	For hierarchical and complex designs, identification of the appropriate level for tests and full reporting of outcomes
☒	☐	Estimates of effect sizes (e.g. Cohen's d, Pearson's r), indicating how they were calculated

Our web collection on statistics for biologists contains articles on many of the points above.

Software and code

Policy information about availability of computer code

Data collection	HE staining image acquisition: KF-PRO-120 scanner (KFBIO); Immunofluorescence staining acquisition: NIKON ECLIPSE C1 system (Nikon Corporation), Pannoramic SCAN II system (3DHISTECH Ltd.); Flow cytometry: FACSCanto II system and FACSFortessa system (BD Biosciences); CyTOF data acquisition: Helio3 CyTOF Mass Cytometer (Fluidigm); scRNA-seq: 10X Chromium Single Cell Platform (Single Cell 5′ library and Gel Bead Kit v.3) , Illumina NovaSeq 6000(Illumina); FASC-sorting: Aria II cell sorter (BD Biosciences); Real-Time PCR: Bio–Rad MyiQ Real-Time PCR Detection System(Bio–Rad).
Data analysis	Flow cytometry: FlowJo software (version 10.0.7); CyTOF data analysis: Phenograp (https://github.com/jacoblevine/PhenoGraph), PARC (https://github.com/ShobiStassen/PARC), Xshif (https://github.com/ginberg/xshift_operator) algorithms, R package cytofkit (version 0.13), pHeatmap R package and Python (https://www.python.org/). scRNA-seq data analysis: CellRanger (version 4.0.0, 10x Genomics) (https://github.com/10XGenomics/cellranger), Seurat v3 R package (http://satijalab.org/seurat/), Monocle (version 2) (http://coletrapnell-lab.github.io/monocle-release/docs/), velocyto.R (version 0.6) (http://velocyto.org), CellPhoneDB (https://www.cellphonedb.org), OmicStudio tools (https://www.omicstudio.cn/tool) ; statistical analysis :GraphPad Prism (v8). Custom codes were not created for data analyses in this study.

For manuscripts utilizing custom algorithms or software that are central to the research but not yet described in published literature, software must be made available to editors and reviewers. We strongly encourage code deposition in a community repository (e.g. GitHub). See the Nature Portfolio guidelines for submitting code & software for further information.

Data

Policy information about availability of data

All manuscripts must include a data availability statement. This statement should provide the following information, where applicable:

- Accession codes, unique identifiers, or web links for publicly available datasets
- A description of any restrictions on data availability
- For clinical datasets or third party data, please ensure that the statement adheres to our policy

The raw scRNA-seq data reported in this paper has been deposited in the Genome Sequence Archive in National Genomics Data Center under the accession number HRA002334 (https://ngdc.cncb.ac.cn/gsa-human/browse/HRA002334). To comply with the "Guidance of the Ministry of Science and Technology (MOST) for the Review and Approval of Human Genetic Resources", raw data can be obtained by request to the corresponding authors and following the guidelines for Genome Sequence Archive for non-commercial use at https://ngdc.cncb.ac.cn/gsa-human/request/HRA002334. There are no time restrictions once access has been granted. The guidance for making a data access request of GSA for humans can be downloaded from https://ngdc.cncb.ac.cn/gsa-human/document/GSA-Human_Request_Guide_for_Users_us.pdf. For analysis of the normal thymus, Jong-Eun Park et al.'s dataset were downloaded from ArrayExpress (accession number E-MTAB-8581;https://www.ebi.ac.uk/arrayexpress/experiments/E-MTAB-8581/). The remaining data were available within the Article, Supplementary Information, or Source Data file. Source data are provided with this paper.

Human research participants

Policy information about studies involving human research participants and Sex and Gender in Research.

Reporting on sex and gender	The design of our study did not consider the effect of gender. The gender of the participants in our study was randomly determined. Our study did not focus on the effect of gender on TET tumors and therefore no sex- and gender-based analyses have been performed.
Population characteristics	Population characteristics are outlined in Supplementary Table 2. Tumor tissues (T, homogeneous cellularity, without necrotic foci) were obtained from patients (56, 69, 72, 55, 67, 44, 31, 68, 58, 37, 66, 68, 35, 60, 61, 63, 50, 25, 73, 48, 68, 58, 61, 63, 57, 61, 49, 41, 55, 63, 42, 36, 35, 55, 51, 51, 46, 49, 45, 58, 63, 50 yo) with TETs who underwent surgical resection at the Department of Thoracic Surgery, Second Affiliated Hospital, Zhejiang University School of Medicine. None of the patients had received radiotherapy or chemotherapy before surgery. The pathological results of all patients indicated TETs. Normal human thymus samples were obtained from the Department of Cardiac Surgery, Second Affiliated Hospital, Zhejiang University following cardiothoracic surgery on adults (27, 46, 34 yo) with heart disease, as thymic tissue is routinely removed and discarded to achieve adequate exposure of the retrosternal operative field. Other characteristics were not used as covariates in the study.
Recruitment	All patients with thymic epithelial tumors that were planed to undergo primary surgery could be asked to be recruited without selection. All patients provided written consent to participate in the study approval of local medical ethnics. There were no self-selection bias or other biases.
Ethics oversight	All samples were anonymously coded in accordance with local ethical guidelines (as stipulated by the Declaration of Helsinki), written informed consent was obtained, and the protocol was approved by the Review Board of the Second Affiliated Hospital of Zhejiang University School of Medicine.

Note that full information on the approval of the study protocol must also be provided in the manuscript.

Field-specific reporting

Please select the one below that is the best fit for your research. If you are not sure, read the appropriate sections before making your selection.

☒ Life sciences ☐ Behavioural & social sciences ☐ Ecological, evolutionary & environmental sciences

For a reference copy of the document with all sections, see nature.com/documents/nr-reporting-summary-flat.pdf

Life sciences study design

All studies must disclose on these points even when the disclosure is negative.

Sample size	In our study, a total of 42 patient samples were included. For CyTOF analysis, we included 22 patient samples and 3 normal samples. For scRNA-seq analysis, we included 6 patient samples and 1 normal samples. A total of 52,788 cells from tumors and 2,845 cells from the normal thymus were included for scRNA-seq analysis. For immuno-fluorescent staining, sections from 28 samples were included in the study.Flow cytometry was performed on 31 patient samples. In vitro co-culture experiment was performed on 6 patients.Sample size both for in vitro and in vivo was chosen taking in consideration the means of the target values between the experimental group and the control group, the standard error and the statistical analysis used. The sample size was able to ensure that our results were reliable.

Data exclusions	All cells expressing <500 genes were removed, as well as cells that contained <500 unique molecular identifiers (UMIs) and >25% mitochondrial counts.
Replication	Experiments were replicated several times with reproducible results as indicated in figure legend/Statistics and reproducibility.
Randomization	Samples were allocated to groups based on disease status (normal and tumor tissue) if applicable. The sample inclusion was random and there was no subjective allocation.
Blinding	In our study, blinding was not applicable to this study since it is exploratory in character and have no elements that might be influenced bybias from the subject or observer.

Reporting for specific materials, systems and methods

We require information from authors about some types of materials, experimental systems and methods used in many studies. Here, indicate whether each material, system or method listed is relevant to your study. If you are not sure if a list item applies to your research, read the appropriate section before selecting a response.

Materials & experimental systems

n/a	Involved in the study	
☐	☒	Antibodies
☒	☐	Eukaryotic cell lines
☒	☐	Palaeontology and archaeology
☒	☐	Animals and other organisms
☒	☐	Clinical data
☒	☐	Dual use research of concern

Methods

n/a	Involved in the study	
☒	☐	ChIP-seq
☐	☒	Flow cytometry
☒	☐	MRI-based neuroimaging

Antibodies

Antibodies used

For immuno-fluorescent staining: Antibodies against CD3 (Clone #SP7, Cat#:ab16669, diluted 1:100), CD8a (Clone #C8/144B, Cat#:ab17147, diluted 1:100), CD4 (Clone #EPR6855, Cat#:ab133616, diluted 1:500), CD103 (Clone #EPR4166(2), Cat#:ab129202, diluted 1:800), CD11c (Clone #EP1347Y, Cat#:ab52632, diluted 1:500), CD20 (Clone #EP459Y, Cat#:ab78237, diluted 1:2000) and EPCAM (Clone #EPR20532-225, Cat#:ab223582, diluted 1:500) were procured from Abcam. Antibodies against CD45 (60287-1-IG, diluted 1:500), CCL25 (25285-1-AP, diluted 1:500), CHI3L1 (12036-1-AP, diluted 1:400), AIRE (22517-1-AP, diluted 1:500), KRT14 (60320-1-IG, diluted 1:800) and GNB3 (12036-1-AP, diluted at 1:400) were procured from Proteintech Group. For flow cytometry:The antibodies CD45-BV510 (Clone #2D1, Cat#:368525, diluted 1:100), CD45-APCCY7 (Clone #HI30, Cat#:304014, diluted 1:100), CD3-BV421 (Clone #UCHT1, Cat#:300433, diluted 1:100), CD3-BV605 (Clone #UCHT1, Cat#:300459, diluted 1:100), CD3-APCCY7 (Clone #UCHT1, Cat#:300425, diluted 1:100), CD3-PECY7 (Clone #UCHT1, Cat#:300420, diluted 1:100), CD4-APC (Clone #RPA-T4, Cat#:300514, diluted 1:100), CD4-PECY7 (Clone #RPA-T4, Cat#:300512, diluted 1:100), CD8a-APCCY7 (Clone #RPA-T8, Cat#:301016, diluted 1:100), CD8a-AF700 (Clone #RPA-T8, Cat#:301028, diluted 1:100), CD8a-FITC (Clone #RPA-T8, Cat#:301006, diluted 1:100), CD103-BV605 (Clone #Ber-ACT8, Cat#:350218, diluted 1:100), CD103-FITC (Clone #Ber-ACT8, Cat#:350204, diluted 1:100), CD69-PECY7 (Clone #FN50, Cat#:310911, diluted 1:100), CD69-PerCP-Cy5.5 (Clone #FN50, Cat#:310925, diluted 1:100), CD39-PE (Clone #A1, Cat#:328207, diluted 1:100), IFN-γ-PE (Clone #B27, Cat#:506506, diluted 1:50), CXCR3-BV421 (Clone #G025H7, Cat#:353715, diluted 1:100), EPCAM-PE (Clone #9C4, Cat#:324205, diluted 1:100), EPCAM-APC (Clone #CO17-1A, Cat#:369809, diluted 1:100), and 7-AAD (Cat#:420404, diluted 1:200) were procured from BioLegend. CXCL13-APC (Clone # 53610, Cat#: IC801A, diluted 1:20) was procured from R&D Systems. HRP conjugated Goat Anti-Mouse/Anti-Rabbit secondary antibodies (ab2891, 1:200) were procured from Abcam.

Validation

All antibodies used in this study were obtained from commercial source, validation information is available from manufactures' websites.
CD3: https://www.abcam.cn/cd3-antibody-sp7-ab16669.html
CD8a: https://www.abcam.cn/cd8-alpha-antibody-c8144b-ab17147.html
CD4: https://www.abcam.cn/cd4-antibody-epr6855-ab133616.html
CD103:https://www.abcam.cn/cd103-antibody-epr41662-ab129202.html
CD11c:https://www.abcam.cn/cd11c-antibody-ep1347y-c-terminal-ab52632.html
CD20:https://www.abcam.cn/cd20-antibody-ep459y-ab78237.html
EPCAM:https://www.abcam.cn/epcam-antibody-epr20532-225-ab223582.html
CD45:https://www.ptgcn.com/products/CD45-Antibody-60287-1-Ig.htm
CCL25:https://www.ptgcn.com/products/CCL25-TECK-Antibody-25285-1-AP.htm
CHI3L1:https://www.ptgcn.com/products/CHI3L1-Antibody-12036-1-AP.htm
AIRE:https://www.ptgcn.com/products/AIRE-Antibody-22517-1-AP.htm
KRT14:https://www.ptgcn.com/products/KRT14-Antibody-60320-1-Ig.htm
GNB3:https://www.ptgcn.com/products/CHI3L1-Antibody-12036-1-AP.htm
HRP conjugated Goat Anti-Mouse/Anti-Rabbit secondary antibodies: https://www.abcam.cn/goat-mouse-rabbit-iggigm-hl-hrp-polymer-ab2891.html
DAPI: https://www.abcam.cn/dapi-ab285390.html
CD45-BV510:https://www.biolegend.com/en-us/products/brilliant-violet-510-anti-human-cd45-antibody-14685
CD45-APCCY7:https://www.biolegend.com/en-us/products/apc-cyanine7-anti-human-cd45-antibody-1914
CD3-BV421:https://www.biolegend.com/en-us/products/brilliant-violet-421-anti-human-cd3-antibody-7153

CD3-BV605:https://www.biolegend.com/en-us/products/brilliant-violet-605-anti-human-cd3-antibody-10421
CD3-APCCY7: https://www.biolegend.com/en-us/products/apc-cyanine7-anti-human-cd3-antibody-3929
CD3-PECY7:https://www.biolegend.com/en-us/products/pe-cyanine7-anti-human-cd3-18693
CD4-APC:https://www.biolegend.com/en-us/products/apc-anti-human-cd4-antibody-823
CD4-PECY7:https://www.biolegend.com/en-us/products/pe-cyanine7-anti-human-cd4-antibody-829
CD8a-APCCY7:https://www.biolegend.com/en-us/products/apc-cyanine7-anti-human-cd8a-antibody-832
CD8a-AF700:https://www.biolegend.com/en-us/products/alexa-fluor-700-anti-human-cd8a-antibody-3396
CD8a-FITC:https://www.biolegend.com/en-us/products/fitc-anti-human-cd8a-antibody-834
CD103-BV605:https://www.biolegend.com/en-us/products/brilliant-violet-605-anti-human-cd103-integrin-alphae-antibody-10362
CD103-FITC:https://www.biolegend.com/en-us/products/fitc-anti-human-cd103-integrin-alphae-antibody-6917
CD69-PECY7:https://www.biolegend.com/en-us/products/pe-cyanine7-anti-human-cd69-antibody-1918
CD69-PerCP-Cy5.5:https://www.biolegend.com/en-us/products/percp-cyanine5-5-anti-human-cd69-antibody-5606
CD39-PE:https://www.biolegend.com/en-us/products/pe-anti-human-cd39-antibody-4364
IFN-γ-PE:https://www.biolegend.com/en-us/products/pe-anti-human-ifn-gamma-antibody-1536
CXCR3-BV421:https://www.biolegend.com/en-us/products/brilliant-violet-421-anti-human-cd183-cxcr3-antibody-7712
EPCAM-PE:https://www.biolegend.com/en-us/products/pe-anti-human-cd326-epcam-antibody-3757
EPCAM-APC:https://www.biolegend.com/en-us/products/apc-anti-human-cd326-epcam-antibody-14168
7-AAD:https://www.biolegend.com/en-us/products/7-aad-viability-staining-solution-1649
CXCL13-APC:https://www.rndsystems.com/cn/products/human-cxcl13-blc-bca-1-apc-conjugated-antibody-53610_ic801a

Flow Cytometry

Plots

Confirm that:

☒ The axis labels state the marker and fluorochrome used (e.g. CD4-FITC).

☒ The axis scales are clearly visible. Include numbers along axes only for bottom left plot of group (a 'group' is an analysis of identical markers).

☒ All plots are contour plots with outliers or pseudocolor plots.

☒ A numerical value for number of cells or percentage (with statistics) is provided.

Methodology

Sample preparation	Freshly excised tissues were stored in sterile RPMI (Corning) supplemented with 10% FBS (Life Technologies) and 1% streptomycin and penicillin (Life Technologies) and processed within 2 hours. The tissues were cut into small pieces and then digested in RPMI containing 10% FBS, type I collagenase (1 mg/ml), and type IV collagenase (1 mg/ml) for 1 hour at 37 °C using a gentleMACSTM Dissociator (Miltenyi Biotech) according to the manufacturer's instructions. The resulting single-cell suspension was filtered sequentially through sterile 70 μm cell strainers. Then, the cell suspensions were stored in complete medium at 4 °C for subsequent experiments.
Instrument	Data were collected on a FACSCanto II system and FACSFortessa system (BD Biosciences) .
Software	Data were analyzed using FlowJo software (version 10.0.7).
Cell population abundance	The abundance of EPCAM+ cells , CD3+CD4+CD8+ cells and CD3-CD4+CD8+ soterd in Suppl. Fig7 h and Suppl. Fig13 was approximately 0.1% in live cells , 60% in CD3+ cells and 60% in CD3- cells, respectively. The purity of all cells after sorting was above 90% as shown in Suppl Fig13.
Gating strategy	The gating used in Fig.1l and Fig.2h has been done as it follows: FSC-H/FSC-A, SSC-A/FSC-A, FSC-H/CD45 (CD45+ cell), FSC-H/CD3(CD3- cell and CD3+ cell), CD4/CD8(CD3-CD4+CD8+ and CD3+CD4+CD8+). The gating strategy used in Fig.6e, i and m has been done as it follows: FSC-H/FSC-A, SSC-A/FSC-A, CD45/CD3 (CD45+CD3+ cell), CD4/CD8 (CD3+CD4+ cell and CD3+CD8+ cell), CD103/CD69 (CD8+CD103+CD69+ TRM and CD8+CD103- non-TRM), FSC-H/CXCR3(IFN-γ/CD39/CXCL13).The gating strategy used in Suppl. Fig.13i-k have been shown Suppl. Fig13.h. The gating strategy used in Suppl. Fig.14a has been done as it follows: FSC-H/FSC-A, SSC-A/FSC-A, FSC-H/CD45 (CD45+ cell), FSC-H/CD3(CD3- cell and CD3+ cell), CD4/CD8(CD3+CD8+), CD103/CD69 (CD8+CD103+CD69+ TRM). Further information are displayed in the figures.

☒ Tick this box to confirm that a figure exemplifying the gating strategy is provided in the Supplementary Information.
